# Predicting Viscosity and Surface Tension at High Temperature of Porcelain Stoneware Bodies: A Methodological Approach

**DOI:** 10.3390/ma11122475

**Published:** 2018-12-06

**Authors:** Sonia Conte, Chiara Zanelli, Matteo Ardit, Giuseppe Cruciani, Michele Dondi

**Affiliations:** 1 CNR-ISTEC, National Research Council-Institute of Science and Technology for Ceramics, Via Granarolo, 64-48018 Faenza (RA), Italy; chiara.zanelli@istec.cnr.it (C.Z.); michele.dondi@istec.cnr.it (M.D.); 2 Physics and Earth Sciences Department, University of Ferrara, Via Saragat, 1-44122 Ferrara, Italy; matteo.ardit@unife.it (M.A.); cru@unife.it (G.C.)

**Keywords:** porcelain stoneware, melt viscosity, melt surface tension, sintering, vitreous phase

## Abstract

The shear viscosity and the glass-vapor surface tension at high temperature are crucial to understand the viscous flow sintering kinetics of porcelain stoneware. Moreover, the pyroplastic deformation depends on the viscosity of the whole body, which is made up of a suspension of crystals dispersed in the melt. The existing fundamental theoretical background, along with semi-empirical constitutive laws for viscous flow sintering and glass densification, can be exploited through different approaches to estimate the physical properties at high temperatures starting from amount and chemical composition of the melt. In this work, a comprehensive attempt to predict the properties of the liquid phase is proposed by means of a detailed overview of existing models for viscosity and surface tension of glasses and melts at high temperature. The chemical composition of the vitreous phase and its physical properties at high temperature are estimated through an experimental approach based on the qualitative and quantitative chemical and phase analyses (by Rietveld refinement of X-ray powder diffraction patterns) of different porcelain-like materials. Repercussions on the firing behavior of ceramic bodies, are discussed. Comparative examples are provided for porcelain stoneware tiles, vitreous china and porcelain bodies, disclosing differences in composition and properties but a common sintering mechanism.

## 1. Introduction

Highly vitrified ceramic tiles—complying the requirements for the Group BI_a_ of ISO 13007—are manufactured essentially with porcelain stoneware bodies. Their chemical composition differs considerably from both porcelain and red stoneware [1,2,3]. The interest on composition and technological behavior of porcelain stoneware bodies has been revitalized by an outstanding innovation process to manufacture gigantic slabs that can exceed 5 square meters [4,5]. In this framework, the physical properties at high temperature of porcelain stoneware tiles are important parameters in monitoring and controlling the different stages of industrial firing. In particular, they play a crucial role in two aspects:

1. The vitrification path, which entails melting of fluxing minerals (primarily feldspars and sericite) and leads to a large amount of vitreous phase, typically ranging from 60 to 75% by weight [6];
2. The kinetics of the viscous flow sintering, which is expected to follow the Frenkel’s and Mackenzie-Shuttleworth’s models, where the densification rate is defined by the glass-vapor surface tension to shear viscosity ratio of the liquid phase [6,7,8].

In addition, the melt viscosity is the main variable accounting for pyroplastic deformation [9,10], as well as any deviation from the expected sintering behavior [11,12].

A serious complication stems from the multiple scale at which the viscosity acts in porcelain stoneware tiles. Actually, the shear viscosity of the liquid phase controls both the melt flow and the rearrangement of the crystal mush at the micrometer scale [13,14]. However, at a macroscopic scale (e.g., firing deformation of a whole slab) the tile viscosity, intended as resistance to high temperature deformation, depends also on the amount, size and shape of crystals dispersed in the melt [15,16].

A fundamental theoretical background of viscous flow sintering already exists and it is based on semi-empirical constitutive laws of glass densification (for an in-depth discussion, the reader is addressed to the specific literature [7,8,16]). Furthermore, predictive models on the viscosity at high temperature, which take into account the chemical composition of the melt, are available for both glasses [17,18] and silicate melts [19,20]. In order to take advantage of these models and to check their applicability to porcelain stoneware tiles, an in-depth knowledge about the chemical composition of the liquid phase at high temperature in porcelain-like bodies is required. On the other hand, the composition of the vitreous phase is seldom investigated in ceramic tiles [6,21] and porcelain [22,23,24]. Further complexity derives from the presence of crystals suspended within the melt for which two different approaches are required:

1. The first aimed at estimating the viscosity of the liquid phase, depending on both its composition and temperature;
2. The second at evaluating the contribution of the solid fraction to the viscosity of the tile as a whole.

Additional variables, such as porosity or size and shape of particles, can play a significant role and should be also considered [12,15].

The present approach does not address the structure of glass network, because the occurrence of a large amount of crystals dispersed in the melt hampers the significance of analyses usually applied to pure glass (for a summary see [25]). This work wants to move a step towards the estimation of the melt viscosity in porcelain-like bodies through its chemical composition. Literature data and new experimental results were used to compare the physical and chemical features of the viscous phase during the sintering process of porcelain stoneware versus vitreous china and porcelain bodies.

## 2. Materials and Methods

Along with the chemical and phase compositions of 7 new porcelain stoneware samples, 45 samples from literature were selected in order to estimate the shear viscosity and the glass-vapor surface tension of the vitreous phase at high temperature (1170–1400 °C). The chemical analyses of the 7 new porcelain stoneware samples were carried out on the fired bodies and their composition was determined by using X-ray fluorescence spectrometry (XRF-WDS, Axios, Malvern Panalytical, Malvern, UK). The standard deviation was between 0.01 and 0.5 wt.%. Since the accurate determination of the phase composition is crucial to a proper estimation of both viscosity and surface tension, only results which derive from quantitative phase analysis through whole-pattern methods (or Rietveld based methods)—which make use of a fixed quantity of a suitably chosen internal standard for the quantification of the vitreous phase—were selected from literature. Specifically, samples can be referred to: 18 porcelain stonewares [10,26,27], 1 vitreous china batch prepared in 20 different conditions [28], 4 hard porcelains and 3 porcelains [29]. The chemical composition of bodies and the phase composition of fired products are reported in Tables S1 and S2, respectively.

**Figure 1:**
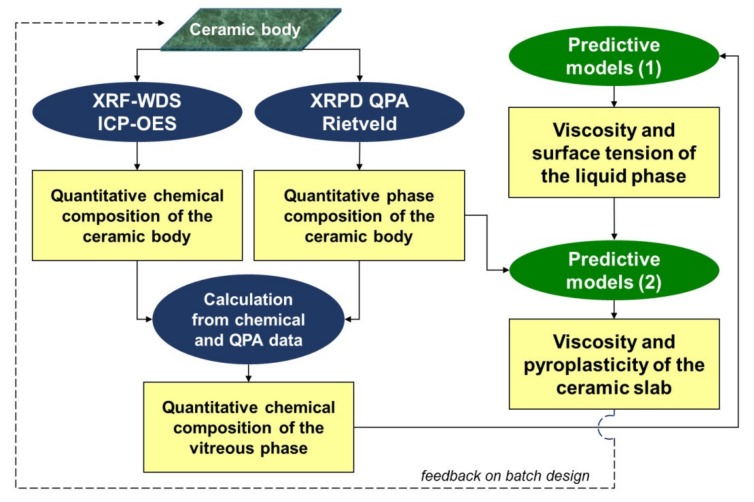
The methodological path followed for the determination of the physical properties of the vitreous phase and porcelain stoneware tile. Predictive models refer to: (1) the liquid phase and (2) the ceramic body as a whole.

The flow-chart of Figure 1 summarizes the methodological path followed, from the chemical and mineralogical composition of ceramic bodies to the determination of both viscosity and surface tension of the vitreous phase. The phase composition at high temperature is determined ex-situ under the assumption that crystals present in the ceramic bodies are all inherited from raw materials or newly formed in the heating step of the firing process. In other words, no crystallization is assumed to occur during cooling. This assumption is proven in most of the experimental cases, because, once the sintering is accomplished, the occurrence of new phases is very uncommon. This is particularly true in ceramic tile manufacturing, where a fast cooling stage (usually few minutes) drops the temperature from ~1200 °C to ~600 °C and crystallization is kinetically prevented. This scenario surely holds for quartz and mullite (where no late crystallization was reported in the literature), even though observations are in some cases doubtful for feldspars [30]. In the case the unit-cell parameters of plagioclase and/or K-feldspar phases deviate significantly from those of feldspathic terms in the batch, an in-situ X-ray diffraction analysis at high temperature (HT-XRD) may be conclusive. However, the accurate reproduction of both powder compaction and the industrial firing schedule (particularly in case of fast firing) with a HT-XRD hot chamber is, at the moment, quite challenging or even impossible. The chemical composition of the vitreous phase was obtained by subtracting the chemical contribution of each mineralogical phase, considering their stoichiometric formula, from the total chemistry of the ceramic bodies. The so obtained values were normalized to 100%.

In this operation, solid solutions should be considered, especially for feldspar and mullite phases. In the vast majority of raw materials used in ceramics, the composition of alkali feldspars is close to that of the end terms, meaning that assuming the albite and orthoclase stoichiometry brings about a tolerable uncertainty related to some small anorthite amount in the plagioclase series and an even smaller albite amount in the case of K-feldspar occurrence. Nevertheless, if it is known from the raw material, the actual feldspar composition can be used for residual crystals. In the case of the mullite phase for the seven new porcelain stoneware samples, its stoichiometry was determined through the Ban & Okada’s empirical model [31]. Indeed, the length of the mullite *a*-axis (nm) depends on the amount of Al_2_O_3_ (in mol %) according to a linear trend that follows the equation:

Al_2_O_3_ = 1443 × *a* − 1028.06 (1)

Therefore, the mullite stoichiometry can be achieved by refining its unit-cell parameters, along with its phase amount.

The calculation of the viscosity of a melt, which depends on both its composition and temperature, has been the subject of several models. Whether developed on silicate glasses or on naturally-occurring silicate melts, all these models are based on the Vogel-Fulcher-Tammann (VTF) equation:

log_10_ η(*T*, *x*) = log_10_ η_∞_(*x*) + A/(*T* − *T*_0_) (2)

where *T* is the temperature, *x* is the composition and the three VFT parameters (η_∞_, A and *T*_0_) are obtained by fitting Equation (2) to experimentally measured viscosity data. The degree of suitability by which models predict the viscosity of melts was checked by a comparison between calculated values and those experimentally measured, taken from literature. In particular, such a comparison was performed for anhydrous silicate melts with composition analogous to those of the vitreous phase in porcelain stoneware [32,33,34,35,36]. The composition of these melts is reported in the Table S3 and results on the viscosity (predicted versus experimental) are illustrated in Figure S1 and Table S4 where Fluegel’s [17] and Giordano and coworkers’ [19] models are compared.

The Fluegel’s model [17] was developed using a global statistical approach coupled with more than 2200 composition–viscosity data for silicate glasses (including soda–lime–silica container and float glasses), TV panel glasses and different type of glasses (e.g., borosilicate and lead crystal glasses, glasses for nuclear waste vitrification, binary alkali silicates and further compositions). This model can be applied to melts characterized by a wide range of compositions, considering that along with silicates it takes into account 54 oxides. Anyway, as well as for some previous attempt [6,21,24], the major drawback derives from the fact that predictive models for the viscosity of glasses (e.g., [16,17]) are formulated on the basis of compositions with a relatively low alumina content. In the case of the liquid phase of porcelain stoneware, the calculation could be performed by extrapolation, thus resulting in a systematic underestimation of viscosity. In fact, data reported in Figure S1 highlight that, by applying the Fluegel’s model, a large discrepancy between calculated and measured viscosity does exist. In particular, experimental data tend to be underestimated. This circumstance may be due to two factors:

(i) The maximum content of alumina considered by the Fluegel’s model (maximum Al_2_O_3_ 19 wt.%) is too low for the systems here in exam (Al_2_O_3_ content from 14.5 to 26.0 wt.%, see Table 1);
(ii) Al is considered to act as a glass network modifier (as in alkali glasses) instead of a glass network former, as it occurs in feldspathic melts [37].

On the other hand, the liquid phase of porcelain stoneware exhibits a remarkable compositional similarity with anhydrous granitic melts. Therefore, it is here suggested that predictive models developed for magmas [19] may be successfully employed to estimate the viscosity at the temperatures which the liquid phase triggers the viscous flow, that is, between 1000 and 1400 °C in porcelain-like bodies. Among all the proposed models, mainly developed on natural silicate melts, that proposed by Giordano, et al. [19] was selected and applied to porcelain-like materials. It was developed on more than 1770 experimentally data on the viscosity of multicomponent anhydrous and volatile-rich silicate melts of known composition. Even if this model takes into account just eleven oxides, it has the advantage to encompass alumina levels closer to those of interest (Al_2_O_3_ up to 23 wt.%). Consequently, experimental and calculated data tend to converge (Figure S1).

The models proposed by Dietzel [38] and Appen [39] were followed to predict the glass-vapor surface tension of the liquid phase at high temperatures. The value at a given temperature was calculated by interpolating the model output that is referred to the surface tension at 900 °C [38] or 1300 °C [39].

## 3. Results and Discussion

### 3.1. Chemical and Phase Composition of Ceramic Bodies

**Figure 2:**
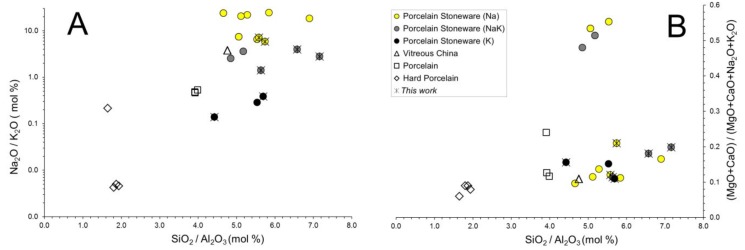
Chemical composition of porcelain stoneware bodies compared with other porcelain-like materials: silica/alumina ratio versus soda/potash ratio (**A**) and alkaline earth oxides to alkaline + alkaline earth oxides ratio (calculated on mol %) (**B**).

In order to make possible a comparison among vitreous phases of the different porcelain-like bodies, an overview of their chemical and mineralogical compositions has been performed. The main compositional features of hard porcelain, porcelain, vitreous china and porcelain stoneware bodies are summarized in Figure 2 and rationalized in terms of the variation of both alkali metal oxides (Na_2_O/K_2_O mol %, Figure 2A) and alkaline earth metal oxides (MgO + CaO/MgO + CaO + Na_2_O + K_2_O mol %, Figure 2B) as a function of the SiO_2_/Al_2_O_3_ ratio (mol %).

Depending on their alkali oxides ratio, porcelain stoneware samples (characterized by a SiO_2_/Al_2_O_3_ ratio between 4.4 and 7.2) can be sorted into three different groups that are: frankly sodic bodies (Na_2_O/K_2_O from 5 up to 24); intermediate sodic-potassic compositions (0.5 < Na_2_O/K_2_O < 5); potassic bodies (Na_2_O/K_2_O < 0.5). In some cases, porcelain stoneware samples, either sodic or sodic-potassic, possess a high amount of MgO and CaO (Figure 2B). This chemical feature indicates the employ of talc, chlorite, dolomite or glass-ceramic frit as sintering promoters [2,10,27]. Other samples belonging to the porcelain stoneware group are also characterized by considerable amount of ZrO_2_ (Table S1) [10]. In contrast with porcelain stoneware, hard porcelain samples have a quite constant chemical composition: mainly potassic, with a SiO_2_/Al_2_O_3_ ratio smaller than 2 and a small amount of alkaline earth oxides. The silica/alumina ratio of both porcelain and vitreous china samples (i.e., SiO_2_/Al_2_O_3_ ~4.4) is comprised between that of the samples above. Albeit represented by just one sample [28], the phase composition of the vitreous china (Table S2) is variable due to the different starting grain size of quartz (d50: 18–50 µm) and also to the firing conditions, that is, temperature (1240–1280 °C) and soaking time (0–20–40–60–80 min).

**Figure 3:**
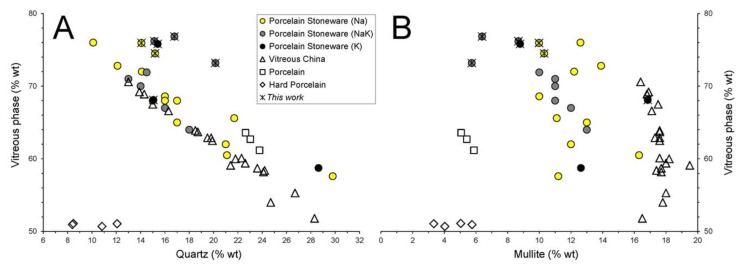
Phase composition of porcelain stoneware bodies compared with other porcelain-like materials: amount of vitreous phase versus quartz (**A**) and mullite (**B**).

The variation of the vitreous phase present in the selected samples is plotted as a function of quartz and mullite (Figure 3). Being the two major components in porcelain-like bodies, regardless of their chemical variability, quartz and vitreous phase are inversely correlated (Figure 3A). This happens also because quartz, once dissolved, turns directly into the vitreous phase.

It should be mentioned that, while the vitreous phase content of vitreous china and porcelain samples never exceeds 70 wt.% and usually ranges from 55 to 65 wt.%, some porcelain stoneware samples are characterized by higher vitreous phase content (i.e., up to 77 wt.%).

Due to a different SiO_2_/Al_2_O_3_ ratio (see both Tables S1 and S2), hard porcelain samples behave as outlier. Indeed, they are characterized by the smallest quantity of both quartz and vitreous phase but the highest quantity of corundum (34 wt.% on average), corresponding to the highest level of alumina of the bodies.

Irrespective of the vitreous phase content, the different typologies of porcelain-like bodies are characterized by a specific mullite amount (Figure 3B). Such different contents likely reflect a diverse batch design, with special care to the amount of kaolinite, which is the source of the primary mullite [40]. In particular, the mullite content in porcelain stoneware is comprised in the 6–16 wt.% range, while in vitreous china it always exceeds 16 wt.% Indeed, kaolinitic clays typically represent 30–40 wt.% of porcelain stoneware batches, while in vitreous china batches they are used in a larger share, 40–50 wt.% of kaolin and ball clays [3]. Thus, a larger mullite content is expected in vitreous china, also because of the higher temperature and longer firing schedule with respect to porcelain stoneware.

### 3.2. Chemical Composition of the Vitreous Phases

The chemical composition of the vitreous phase is reported in Table 1. The reliability of results strongly depends on the phase composition accuracy. For this reason, only works with a proper application of full profile refinements of XRPD patterns were selected. The chemical composition of the different vitreous phases was obtained through the methodological path described in the experimental approach. This quantitative approach is here applied for the first time to get the composition of the liquid phase in porcelain stoneware, along with a systematic review of ceramic tiles.

The chemistry of the vitreous phases pertaining to the different porcelain-like materials somehow reflects the chemical composition of the starting bodies. Such vitreous phases are characterized by high, though variable in rather wide ranges, contents of silica and alumina (65–77 wt.% SiO_2_, 14–26 wt.% Al_2_O_3_, Table 1). In addition, both hard porcelain and porcelain exhibit a potassic signature, at variance of porcelain stoneware and vitreous china, which are characterized by higher Na_2_O/K_2_O ratios. The variable amounts of silica and alumina depend on the persistence of quartz and mullite in contact with the melt in a dynamic equilibrium. In fact, the crystalline phases act as buffers of silica (quartz) and alumina plus to a lesser extent silica (mullite). The same role is played, even though on a smaller scale, by zircon and rutile, which buffer ZrO_2_ and TiO_2_, respectively [41,42]. The dissolution of quartz is known to take place with an increasingly faster rate over 1150 °C. This fact brings about a progressive enrichment in silica of the liquid phase. On the other hand, the dissolution of mullite—usually a sluggish phenomenon—leads to peraluminous melts. Both these mechanisms can significantly affect the viscosity and surface tension of the liquid phases [6,17,43].

**Figure 4:**
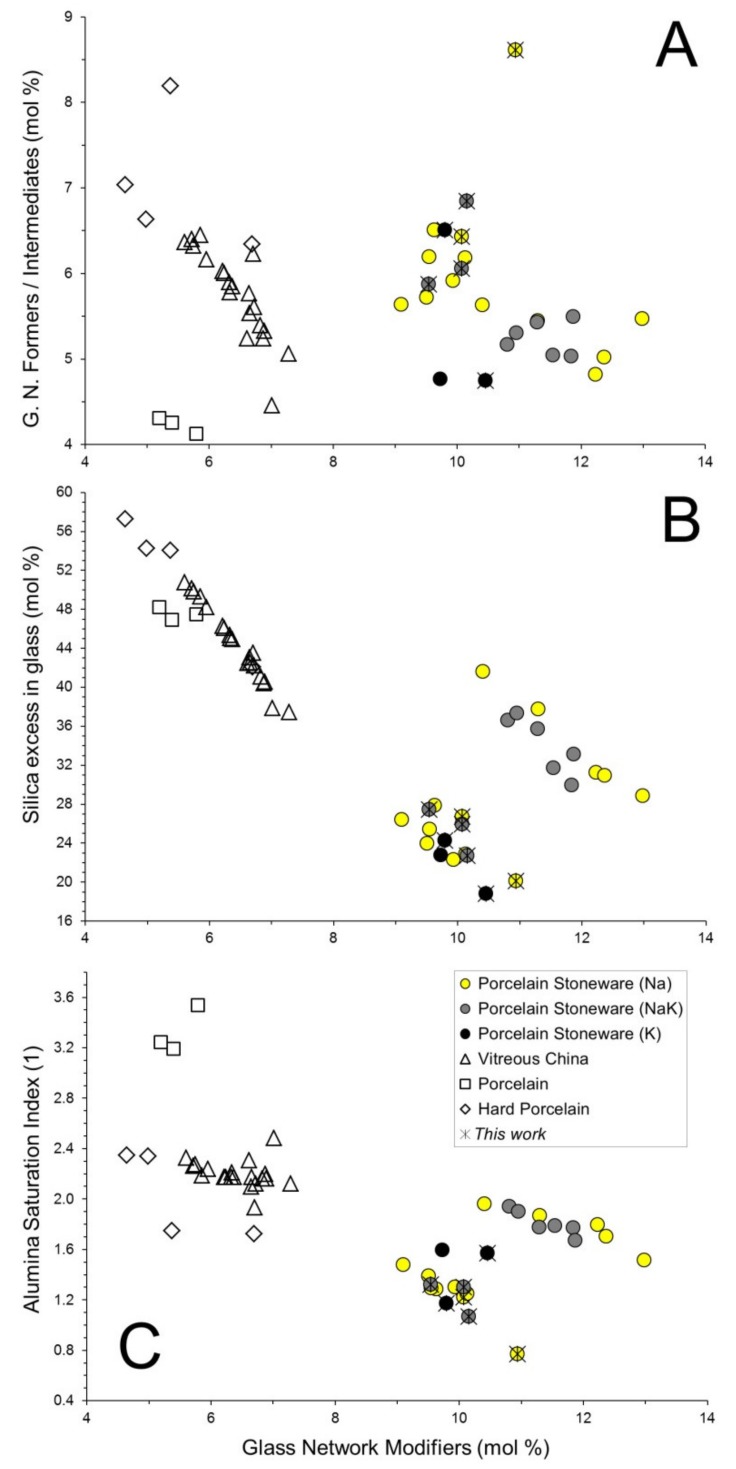
Chemical composition of the vitreous phase in porcelain stoneware bodies compared to other porcelain-like materials. Glass Network Modifiers (MgO + CaO + Na_2_O + K_2_O) versus (**A**) the ratio of Glass Network Formers (SiO_2_) to Intermediates (TiO_2_ + Al_2_O_3_ + Fe_2_O_3_ + ZrO_2_); (**B**) silica in excess with respect to a feldspathic melt; (**C**) Alumina Saturation index calculated on mol % (Al_2_O_3_/CaO + Na_2_O + K_2_O).

Interestingly, the alumina percentage in the melt is higher in porcelain than in vitreous china and porcelain stoneware. Hard porcelain seems to have a peculiar melt composition, that is, a silica enrichment and an alumina deficiency, with respect to the conventional porcelain. Another fundamental factor is the amount of Glass Network Modifiers (GNM = MgO + CaO + Na_2_O + K_2_O) that in porcelain-like materials are essentially represented by alkaline and alkaline-earth oxides. GNM are most abundant in porcelain stoneware, followed by vitreous china and porcelain, while the ratio between Glass Network Formers (SiO_2_) and Intermediates (TiO_2_ + Al_2_O_3_ + Fe_2_O_3_ + ZrO_2_) is usually in between 5 and 7 in porcelain stoneware and vitreous china (Table 1, Figure 4A).

The composition of the vitreous phase can be represented in the SiO_2_-NaAlSi_3_O_8_-KAlSi_3_O_8_ system, since it is mainly the result of feldspars and quartz breakdown. From the kinetic viewpoint, feldspars melting occurs very rapidly, starting usually around 1050 °C, while the quartz dissolution is a sluggish reaction, being activated usually over 1150 °C [6]. By this way, it is possible to assess the quartz contribution to the formation of the vitreous phase, expressed as excess of silica, Si(ex), that is the SiO_2_ percentage in the vitreous phase exceeding that derived by the feldspar stoichiometry and some stemming from clay minerals breakdown. Such an excess ranges from ~20% mol in porcelain stoneware up to ~60% mol in hard porcelain (Figure 4B). The inverse correlation between GNM and Si(ex) suggests that higher content of network modifiers (larger amount of feldspars in porcelain stoneware) entail a lesser involvement of quartz in the formation of the vitreous phase and vice versa in vitreous china and porcelain.

**Figure 5:**
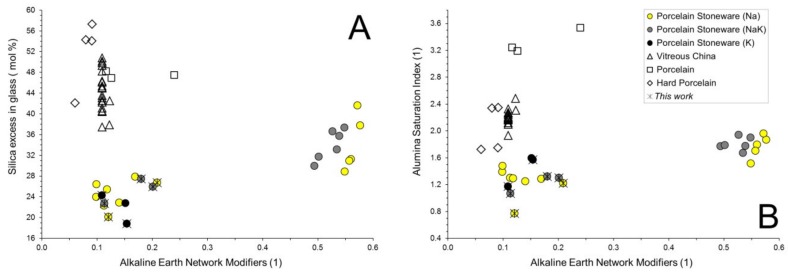
Chemical composition of the vitreous phase in porcelain-like materials. Fraction of alkaline earth on the total amount of Glass Network Modifiers calculated on mol % (MgO + CaO/MgO + CaO + Na_2_O + K_2_O) versus (**A**) silica in excess with respect to a feldspathic melt and (**B**) Alumina Saturation index (calculated on mol %).

Along with these evidences, in the specific case of porcelain and hard porcelain, high values of Si(ex) are also related to firing temperatures which can reach 1300 °C and 1400 °C, respectively. With regards to the porcelain stoneware, two parallel alignments clearly arise in Figure 4B, implying a somehow variable capacity to dissolve quartz, only partially justified by the different amounts of GNM. This seems to be induced by alkaline earth oxides: indeed, the porcelain stoneware bodies richer in MgO and CaO exhibit a larger excess of silica in the melt (Figure 5A). This observation suggests that a vitreous phase where alkaline earth oxides represent a large fraction of GNM tends to coexist with some feldspars and a lower amount of quartz (as residual crystalline phases). Anyway, this is just a preliminary result that needs to be verified by further experimental work.

On the other hand, the involvement of clay minerals (and their transformation products, e.g., mullite) in the vitrification process is proved by the Alumina Saturation Index (ASI) that expresses the Al_2_O_3_ exceeding the albite-anorthite-orthoclase stoichiometry (ASI = 1 for a pure feldspathic melt). In porcelain stoneware the ASI values are between 1 and 2, while vitreous china and hard porcelain plot in the 1.7–2.5 range (Figure 4C). The higher the ASI, the larger the amount of alumina in the melt not supplied by feldspars. Thus, this parameter is somewhat inversely related to the mullite stability, which would increase from vitreous china and hard porcelain towards the porcelain stoneware with a low fraction of alkaline earth oxides (1 < ASI < 1.5). In fact, porcelain stoneware bodies rich in MgO and/or CaO exhibit higher ASI, in between vitreous china and classic porcelain stoneware (Figure 5B).

### 3.3. Physical Properties at High Temperature of the Vitreous Phases

The physical properties of the liquid phase—as predicted at the typical firing temperature (*T*_max_) for every porcelain-like material—are reported in Table 2. On the whole, the data range from 4.25 to 5.35 log_10_ Pa·s for shear viscosity and from 292 to 360 mN·m^−1^ for glass-vapor surface tension. The temperature of glass transition fluctuates in between 716 and 823 °C.

**Figure 6:**
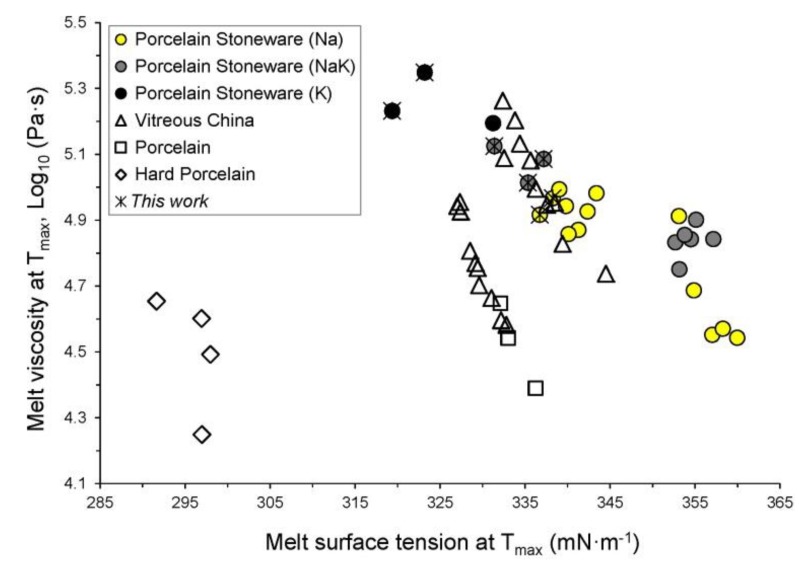
Viscosity versus surface tension at the maximum firing temperature (*T*_max_) of the vitreous phases.

Surface tension data are attested in rather narrow ranges for every typology but porcelain stoneware, which values are in between 315 and 360 mN·m^−1^. This variability is about 13% of the average (344 mN·m^−1^). The surface tension values of hard porcelain never exceed 300 mN·m^−1^. On the contrary, the range of melt viscosity is always wide, particularly for porcelain stoneware (4.5–5.4 log_10_ Pa·s) whose range represents approximately two times the average value (Figure 6). For this reason, the melt viscosity plays a predominant role in viscous flow sintering.

**Figure 7:**
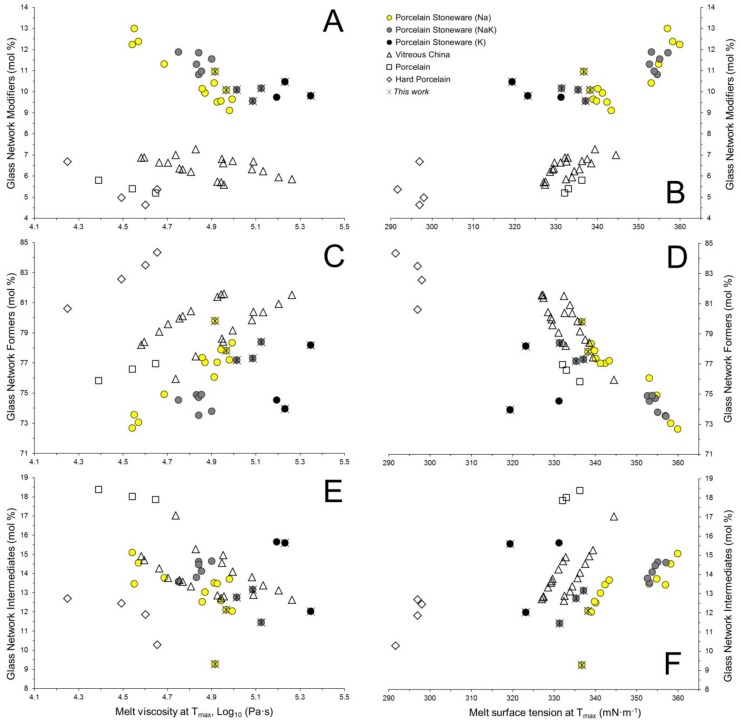
Physical properties at the maximum firing temperature (*T*_max_) of the vitreous phase in function of its chemical composition. Glass Network Modifiers versus viscosity (**A**) and surface tension (**B**); Glass Network Formers versus viscosity (**C**) and surface tension (**D**); Glass Network Intermediates versus viscosity (**E**) and surface tension (**F**).

Since alkaline and alkaline-earth oxides have fluxing properties that lead to the formation of non-bridging oxygen sites, the GNM amount inversely scales with the melt viscosity (Figure 7A). Albeit the GNM increasing seems to be related to an increase of surface tension (Figure 7B), this fact can be translated as the occurrence of three coexisting evidences: (i) porcelains and vitreous china scale directly with the GNF/GNI ratio; (ii) porcelain stoneware shows an increasing surface tension with the Na_2_O/K_2_O ratio; (iii) the highest values are attained in porcelain stoneware rich in alkaline earth oxides.

Silica is the main Glass Network Former in the materials under investigation. It is directly correlated with the melt viscosity (Figure 7C) and inversely correlated with surface tension (Figure 7D). The different typologies in Figure 7C describe parallel trends that, for a given amount of GNF (e.g., the mean value 78 mol %), can be interpreted in the following order: hard porcelain < vitreous china ~ porcelain < Na < Na-K < K porcelain stoneware. Parallel to silica, alumina is the main component among the Glass Network Intermediates. However, Al_2_O_3_ can act either as glass network former or modifier in function of its oxygen coordination number (from 4 to 5 and 6), depending on the relative proportions with the other oxides, as demonstrated by spectroscopic analyses on feldspathic and peraluminous glasses [2,37,44,45,46]. Apparently, the GNI seem to lower the melt viscosity, even though by a gentle slope (Figure 7E). This trend is convoluting various effects and not merely the variation of the alumina content. It is important to stress that Al_2_O_3_ is strongly correlated to alkali in porcelain stoneware bodies; so, any alumina increasing in the melt implies an enrichment in alkali too. Therefore, the tendency to lower the melt viscosity is likely to be a side effect of the contemporaneous increasing in GNM. Conversely, surface tension presents a steep and direct correlation with the GNI (Figure 7F).

**Figure 8:**
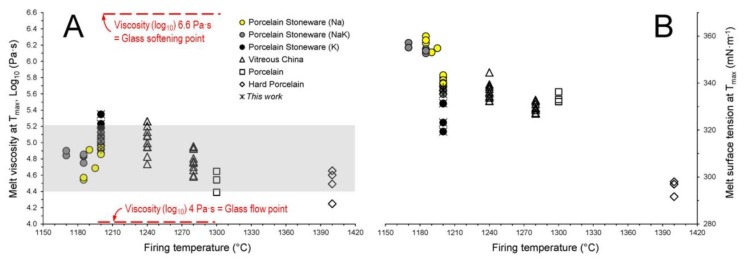
Correlation of viscosity (**A**) and surface tension (**B**) of the vitreous phase versus the firing temperature of the melt in porcelain stoneware compared with other porcelain-like materials.

The firing temperature is contrasted with the viscosity (Figure 8A) and surface tension (Figure 8B) of the vitreous phase. The melt viscosity falls always within a rather narrow range (i.e., from 4.4 to 5.2 log_10_ Pa·s) despite the four porcelain-like types are fired at different maximum temperatures. This is because of the different chemical composition of the vitreous phases, especially with regard to the amount of GNM that in porcelain stoneware is much higher with respect to vitreous china and particularly hard porcelain. Therefore, it seems that the viscous flow sintering occurs, irrespective of the firing temperature, in a critical window of melt viscosity (Figure 8A). Such a viscosity is at least 2.5 times higher than the glass flow point (i.e., 4.4 vs. 4.0 log_10_ Pa·s) while approximately 25 times lower than the glass softening point (i.e., 5.2 vs. 6.6 log_10_ Pa·s).

The surface tension is usually in the range between 320 and 345 mN·m^−1^ (Figure 8B). Exceptions are the hard porcelain on one side and the alkaline earth porcelain stoneware on the other side.

### 3.4. Repercussions on Firing Behavior

It can be observed that, in order to control the viscous flow sintering, a balanced ratio between the amount of vitreous phase and its viscosity is crucial. Specifically, in ceramic bodies where the percentage of vitreous phase is low, as in hard porcelain, a lower viscosity is needed. On the contrary, where the amount of vitreous phase is abundant, as in porcelain stoneware and vitreous china, a higher viscosity to accomplish the densification in the industrial firing schedules is required.

It is important to keep in mind that all these results are only related to the vitreous phase of the porcelain-like bodies, whereas the occurrence of crystals suspended in the melt (the so-called “skeleton” or solid load, ϕ) turns it into a suspension (or a “viscous phase”) rather than a liquid phase. The actual viscosity of this suspension is expected to be strongly affected by the amount, size and shape of microcrystals dispersed in the liquid phase [10,14,15,47]. In addition, a strong deviation from linearity in the solid load-to-viscosity relationship could occur with a critical fraction of solid load (ϕ*_cr_*), which is defined as “the fraction of solid that delimits the transition from a system where the viscosity of the suspension is controlled by the viscosity of the liquid phase, to a system where particle-particle interactions induce a strong increase of viscosity” [47].

The effective viscosity of the melt + crystals system (η*_eff_*) is therefore higher than the viscosity of the liquid phase (η), as here predicted through the Giordano and coworkers’ model [19]. The difference is defined as the relative viscosity:

(3) $$\eta_{rel}=\eta_{eff}-\eta$$

The relative viscosity (η*_rel_*), which is the contribution of the solid fraction to the total viscosity of the system, can be estimated by the Krieger and Dougherty [48] equation:

(4) $$\eta_{rel}=\;{\left(1-\frac{\phi }{\phi_{cr}}\right)}^{-B\;\phi_{cr}}$$

where ϕ is the solid load, ϕ*_cr_* is the critical fraction and *B* is the Einstein constant (i.e., *B* = 2.5). The critical fraction is assumed to be equal to the relative maximum packing density (ϕ*_cr_* = 0.74). For sake of simplicity, here it is assumed that particles are spherical and of the same dimension, so disregarding possible differences in size and shape of crystals.

**Figure 9:**
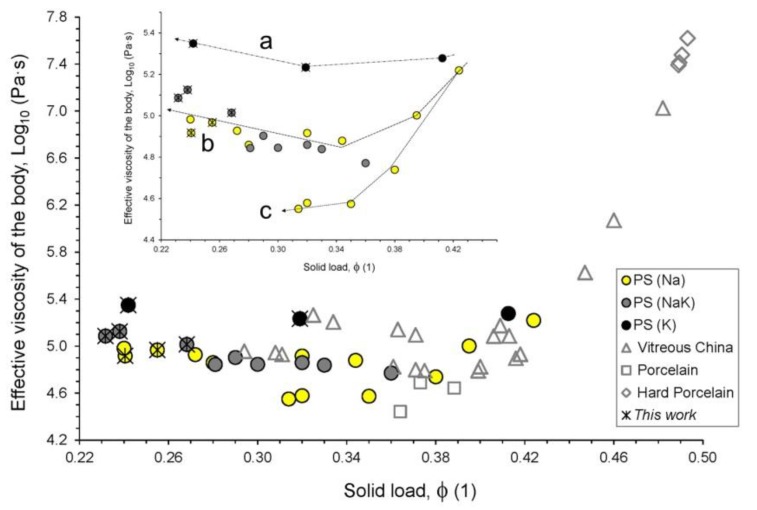
Effective viscosity of the ceramic bodies in function of its solid load (normalized to 1). About porcelain stoneware, three different trends can be seen (see inset, lines are reader’s eye guides): (**a**) potassic bodies; (**b**) sodic and sodic-potassic bodies; (**c**) sodic bodies with high (MgO + CaO)/GNM ratio.

Plotting the effective viscosity as a function of the solid load, the expected trend arose (Figure 9). In detail for porcelain stoneware, three distinct trends are defined (lines a, b and c in the inset of Figure 9). Most samples define two saddle-like, parallel trends: (a) for potassic bodies and (b) for sodic and sodic-potassic bodies. These saddles are characterized by an initial viscosity reduction for decreasing solid load (from 43 wt.% down to 32–34 wt.%) and then a viscosity increasing for a further decrease in solid load (down to 23–24 wt.%).

Although the viscosity drop associated to the reduction of the “skeleton” is expected, the countertrend is not explainable by a mere restraint of shape and size imposed to the particles dispersed in the melt. Thus, the effective viscosity is boosted by a viscosity increase of the liquid phase, due to the growing amounts of silica and alumina provided by the dissolution of quartz and mullite during firing.

The third trend (line c in Figure 9) is represented by some samples whose buffering effect is much less efficient, leading to η*_eff_* values that are approximately one half of those along the trend of line b. We are dealing with sodic bodies with a high amount of alkaline earth oxides, also characterized by a liquid phase with the highest values of Si_(ex)_ and ASI among porcelain stoneware. Since alkaline earth oxides play a strong fluxing role, the relatively high percentages of MgO and CaO somewhat compensate and overcome the opposite effect of “free” silica and alumina. So, the net result is a lowering of the effective viscosity.

## 4. Conclusions

The physical properties at high temperature of the liquid phase present in porcelain stoneware bodies can be inferred through a methodological approach which entails a normalized difference between chemistry of the bulk and their phase composition. Such a quantitative estimation gives back both the shear viscosity and the glass-vapor surface tension and it takes place by applying models from literature based on the chemical composition of the liquid phase. In this contribution it has been demonstrated that satisfactory results are obtained when the models of Giordano, et al. [19] for shear viscosity, as well as those of Dietzel and Appen for surface tension [38,39] are used.

This method represents, beyond any accuracy limit stemming from the quality by which the chemical and phase composition are determined, a simple tool to approach the inner workings of porcelain stoneware sintering. In fact, this process is expected to run by viscous flow, which is essentially governed by the shear viscosity to surface tension ratio. These are the basic properties driving the densification kinetics, which cannot be easily obtained by alternative methods.

In addition, the dimensional stability at high temperature of ceramic wares, once the maximum density is achieved, depends on the effective viscosity of the whole body, intended as a suspension of crystals dispersed in the melt. Such an effective viscosity can be estimated as well, based on the amount and shear viscosity of the liquid phase, by models based on the geometric constraints of solid particles in the melt, as developed for silicate melts [15]. This piece of information is fundamental to explaining and controlling the pyroplasticity of porcelain stoneware, which turned out to be a critical issue with the production of large slabs. In fact, as observed, the viscosity drop associated to the reduction of the “skeleton” is hindered by a viscosity increase of the liquid phase, due to the growing amounts of silica and alumina provided by the dissolution of quartz and mullite during firing. This buffering effect allows to manufacture products of ever larger dimensions with tolerable permanent deformations, as it happens for porcelain stoneware slabs (and vitreous china sanitary wares).

An important outcome is represented by the feedback on batch design: for instance, the effect of a new raw material on sintering kinetics and pyroplasticity can be predicted, accounting for its behavior (as “flux” or “filler”) during firing.

Such an approach proved to be applicable to porcelain as well. Although the present work reports just a few samples, interesting differences about the solid load and melt composition arose between various porcelain-like materials. Nevertheless, these dissimilarities give rise to a common range of melt viscosity during the viscous flow process (i.e., 4.4–5.2 log_10_ Pa·s) besides the firing temperatures are largely different from hard porcelain to vitreous china, down to porcelain stoneware.

## Figures and Tables

**Table 1:** Amount and chemical composition of the vitreous phase in porcelain-like bodies.

wt.%	Sample	Amount	SiO_2_	TiO_2_	ZrO_2_	Al_2_O_3_	Fe_2_O_3_	MgO	CaO	Na_2_O	K_2_O	Calculated From
Porcelain stoneware	1200	58.7	64.9	0.7	—	21.2	1.5	0.4	0.6	1.7	8.8	[26]
	STD.6	65.0	67.7	0.5	—	19.6	1.3	2.9	2.1	4.9	1.1	[10]
	QF	62.0	68.6	0.5	0.5	19.6	1.3	2.8	1.7	3.8	1.0	
	QM	68.0	69.6	0.5	1.0	19.1	1.2	2.5	1.5	3.6	0.9	
	KM	70.0	66.7	0.4	—	21.2	1.2	2.5	1.5	3.9	2.6	
	QFKM	64.0	68.1	0.5	0.5	19.3	1.3	2.7	1.7	4.0	1.9	
	QMKM	67.0	68.2	0.5	1.0	19.4	1.2	2.6	1.6	3.7	1.8	
	STD.6	68.6	65.9	0.5	1.0	21.3	1.2	2.8	2.0	4.4	1.0	
	STD.9	68.0	66.5	0.5	1.0	20.5	1.3	2.8	2.0	4.5	1.0	
	KM	71.0	66.6	0.4	1.0	20.6	1.2	2.4	1.5	3.7	2.5	
	QFKM	71.9	67.7	0.4	0.9	20.4	1.2	2.4	1.5	3.7	1.7	
	QMKM	68.0	68.1	0.5	1.0	19.9	1.2	2.5	1.6	3.5	1.8	
	Na	75.9	70.3	0.9	—	18.2	0.6	0.4	0.5	7.5	1.6	This Work
	NaB	74.5	71.4	0.3	—	17.9	0.8	0.6	1.0	6.4	1.7	
	AT	76.7	70.8	0.6	—	18.3	0.8	0.3	1.0	5.9	2.3	
	ATP	72.9	70.8	0.5	—	17.8	0.8	0.7	0.8	5.5	3.1	
	NaK	76.2	68.7	0.9	—	19.0	0.6	0.4	0.4	4.8	5.2	
	K	75.8	68.2	0.8	—	18.6	0.6	0.4	0.4	2.2	8.9	
	KB	68.1	67.3	0.3	—	18.3	0.8	0.7	0.4	1.0	11.2	
	2	72.8	70.2	0.4	—	19.9	0.6	0.1	0.6	7.8	0.4	[27]
	7	76.0	70.2	0.4	—	20.3	0.5	0.1	0.6	7.5	0.4	
	21	65.6	70.4	0.4	—	19.3	0.6	0.2	0.7	8.1	0.4	
	22	72.0	71.0	0.5	—	18.5	0.6	0.4	0.7	7.9	0.5	
	24	57.6	72.2	0.5	—	17.8	0.6	0.5	0.7	7.3	0.5	
	25	60.5	71.4	0.5	—	18.5	0.7	0.2	0.7	7.7	0.4	
Vitreous china	0-1240-50	51.8	67.2	0.5	—	24.1	1.3	0.3	0.3	4.3	2.0	[28]
	20-1240-50	55.3	69.3	0.5	—	21.8	1.2	0.3	0.2	4.7	1.9	
	40-1240-50	58.7	70.7	0.5	—	20.9	1.1	0.3	0.2	4.5	1.8	
	60-1240-50	59.4	71.4	0.5	—	20.3	1.1	0.3	0.2	4.4	1.8	
	80-1240-50	59.1	73.1	0.5	—	18.6	1.1	0.3	0.2	4.4	1.8	
	0-1280-50	58.4	70.2	0.5	—	21.4	1.1	0.3	0.2	4.5	1.8	
	20-1280-50	58.2	70.5	0.5	—	21.1	1.1	0.3	0.2	4.5	1.8	
	40-1280-50	60.1	71.3	0.4	—	20.6	1.1	0.3	0.2	4.4	1.7	
	60-1280-50	62.5	72.4	0.4	—	19.8	1.0	0.3	0.2	4.2	1.7	
	80-1280-50	62.9	72.6	0.4	—	19.7	1.0	0.3	0.2	4.2	1.7	
	0-1240-18	54.0	70.3	0.5	—	21.4	1.2	0.3	0.2	4.1	1.9	
	20-1240-18	62.9	72.2	0.4	—	20.0	1.0	0.3	0.2	4.2	1.7	
	40-1240-18	63.7	72.9	0.4	—	19.4	1.0	0.3	0.2	4.1	1.6	
	60-1240-18	66.6	73.6	0.4	—	19.1	1.0	0.3	0.2	3.9	1.6	
	80-1240-18	67.5	74.3	0.4	—	18.4	1.0	0.3	0.2	3.9	1.6	
	0-1280-18	60.0	72.0	0.4	—	19.9	1.1	0.3	0.2	4.4	1.7	
	20-1280-18	63.9	73.0	0.4	—	19.3	1.0	0.3	0.2	4.1	1.6	
	40-1280-18	68.9	74.1	0.4	—	18.8	0.9	0.2	0.2	3.8	1.5	
	60-1280-18	69.2	74.3	0.4	—	18.6	0.9	0.2	0.2	3.8	1.5	
	80-1280-18	70.6	74.3	0.4	—	18.7	0.9	0.2	0.2	3.7	1.5	
Porcelain	S5	62.7	67.4	0.9	—	25.1	0.6	—	0.5	1.4	4.1	[29]
	S6	61.2	66.9	0.5	—	25.5	0.8	—	0.6	1.3	4.4	
	S7	63.6	66.3	0.6	—	26.0	0.8	0.5	0.5	1.3	4.1	
Hard porcelain	S1	51.1	74.1	0.6	—	17.8	0.8	—	0.3	—	6.5	[29]
	S2	50.7	75.4	0.7	—	16.7	0.9	—	0.4	—	6.0	
	S3	51.1	76.7	0.7	—	14.5	0.8	—	0.4	—	7.0	
	S4	50.9	72.0	0.9	—	17.5	0.9	—	0.3	1.0	7.2	

**Table 2:** Physical Properties at *T*_max_ of the vitreous phase in porcelain-like bodies.

Porcelain Stoneware	*T*_max_	Viscosity	Surface Tension	*T_g_*, Glass Transition	Sample		*T*_max_	Viscosity	Surface Tension	*T_g_*, Glass Transition
	°C	log_10_ Pa·s	mN·m^−1^	°C			°C	log_10_ Pa·s	mN·m^−1^	°C
1200	1200	5.19	331.2	761	Vitreous China	0-1240-50	1240	4.74	344.5	769
STD.6	1185	4.55	357.0	727		20-1240-50	1240	4.83	339.4	762
QF	1195	4.69	354.9	744		40-1240-50	1240	4.95	337.6	767
QM	1190	4.91	353.1	752		60-1240-50	1240	5.00	336.3	768
KM	1170	4.84	357.1	739		80-1240-50	1240	5.09	332.5	767
QFKM	1185	4.75	353.2	737		0-1280-50	1280	4.58	332.8	767
QMKM	1185	4.83	352.7	743		20-1280-50	1280	4.60	332.2	767
STD.6	1185	4.54	360.0	736		40-1280-50	1280	4.66	331.0	769
STD.9	1185	4.57	358.3	734		60-1280-50	1280	4.75	329.4	773
KM	1170	4.9	355.1	741		80-1280-50	1280	4.77	329.2	774
QFKM	1185	4.84	354.5	747		0-1240-18	1240	4.95	338.5	772
QMKM	1185	4.86	353.8	748		20-1240-18	1240	5.08	335.6	774
Na	1200	4.92	336.7	719		40-1240-18	1240	5.13	334.4	775
NaB	1200	4.97	338.3	727		60-1240-18	1240	5.20	333.8	779
AT	1200	5.09	337.2	737		80-1240-18	1240	5.26	332.4	781
ATP	1200	5.01	335.3	730		0-1280-18	1280	4.70	329.6	769
NaK	1200	5.13	331.4	738		20-1280-18	1280	4.81	328.5	775
K	1200	5.35	323.2	752		40-1280-18	1280	4.93	327.4	782
KB	1200	5.23	319.4	742		60-1280-18	1280	4.94	327.0	783
2	1200	4.93	342.4	726		80-1280-18	1280	4.96	327.3	785
7	1200	4.98	343.4	733	Porcelain	S5	1300	4.65	332.1	808
21	1200	4.87	341.3	719		S6	1300	4.54	333.0	802
22	1200	4.86	340.1	716		S7	1300	4.39	336.2	798
24	1200	4.99	339.0	726	Hard porcelain	S1	1400	4.49	298.0	817
25	1200	4.94	339.8	722		S2	1400	4.60	296.9	823
						S3	1400	4.65	291.6	814
						S4	1400	4.25	297.0	790

## Supplementary Materials

The following are available online at http://www.mdpi.com/1996-1944/11/12/2475/s1, Figure S1: Viscosity predicted versus experimental: Giordano and coworkers’ model (left) and Fluegel’s model (right). The viscosity interval of interest for porcelain-like materials is in between 4 and 6 Log10 Pa·s. IGC [33], MNV [33], MST [34], CI_OF [34], MDV [34], G.2000 [32], AMS-B1 [35], AMS-D1 [35], Trachyte [36], Phonolite [36]. Table S1: Chemical composition of porcelain stoneware bodies in comparison with hard porcelain, porcelain, and vitreous china batches; Table S2: Phase composition of porcelain stoneware bodies in comparison with hard porcelain, porcelain, and vitreous china batches; Table S3: Chemical composition of silicate melts taken into account to assess the Giordano and coworkers’ [19] and Fluegel’s [17] models used to calculate the viscosity at high temperature; Table S4: Comparison between the calculated and the experimentally measured viscosity values of silicate melts.
