# Supplementary material for: Predicting Viscosity and Surface Tension at High Temperature of Porcelain Stoneware Bodies: A Methodological Approach
Source: Materials (Basel). 2018 Dec 6;11(12):2475. doi: 10.3390/ma11122475 (PMC6317026; doi:10.3390/ma11122475)
Supplement: Supplementary file 1 [file materials-11-02475-s001.pdf]

materials

MDPI

Supplementary

Predicting Viscosity and Surface Tension at High Temperature of Porcelain Stoneware Bodies: A Methodological Approach

Sonia Conte [1,*], Chiara Zanelli [1], Matteo Ardit [2], Giuseppe Cruciani [2] and Michele Dondi [1]

[1] CNR-ISTEC, National Research Council-Institute of Science and Technology for Ceramics, Via Granarolo, 64-48018 Faenza (RA), Italy; chiara.zanelli@istec.cnr.it (C.Z.); michele.dondi@istec.cnr.it (M.D.)

[2] Physics and Earth Sciences Department, University of Ferrara, Via Saragat, 1-44122 Ferrara, Italy; matteo.ardit@unife.it (M.A.); cru@unife.it (G.C.)

* Correspondence: sonia.conte@istec.cnr.it; Tel.: +39-0546-699777

Received: 12 October 2018; Accepted: 1 December 2018; Published: 6 December 2018

Table S1 Chemical composition of porcelain stoneware bodies in comparison with hard porcelain, porcelain, and vitreous china batches.

Material	Sample	SiO_2	TiO_2	ZrO_2	Al_2O_3	Fe_2O_3	MgO	CaO	Na_2O	K_2O	Ref.
Porcelain stoneware	1200	70.3	0.4	—	21.5	0.9	0.2	0.4	1.0	5.2	[26]
	STD.6	67.0	0.3	2.1	22.4	0.9	1.9	1.4	3.4	0.7	[10]
	QF	69.4	0.3	2.1	21.3	0.8	1.7	1.1	2.7	0.6	
	QM	69.4	0.3	2.1	21.3	0.8	1.7	1.1	2.7	0.6	
	KM	66.1	0.3	2.1	23.1	0.8	1.7	1.1	3.0	1.8	
	QFKM	67.8	0.3	2.1	22.2	0.8	1.7	1.1	2.8	1.2	
	QMKM	67.8	0.3	2.1	22.2	0.8	1.7	1.1	2.8	1.2	
	STD.6	67.0	0.3	2.1	22.4	0.9	1.9	1.4	3.4	0.7	
	STD.9	67.0	0.3	2.1	22.4	0.9	1.9	1.4	3.4	0.7	
	KM	66.1	0.3	2.1	23.1	0.8	1.7	1.1	3.0	1.8	
	QFKM	67.8	0.3	2.1	22.2	0.8	1.7	1.1	2.8	1.2	
	QMKM	67.8	0.3	2.1	22.2	0.8	1.7	1.1	2.8	1.2	
	Na	70.0	0.7	—	21.3	0.5	0.3	0.4	5.7	1.2	This Work
	NaB	71.0	0.2	—	21.0	0.6	0.4	0.8	4.8	1.3	
	AT	72.7	0.6	—	18.8	0.6	0.2	0.8	4.5	1.7	
	ATP	73.9	0.5	—	17.5	0.6	0.5	0.6	4.2	2.3	
	NaK	69.7	0.7	—	21.0	0.5	0.3	0.3	3.7	4.0	
	K	69.3	0.6	—	20.7	0.4	0.3	0.3	1.7	6.7	
	KB	65.2	0.2	—	25.0	0.6	0.5	0.3	0.7	7.6	
	2	67.9	0.3	—	24.7	0.4	0.1	0.5	5.8	0.4	[27]
	7	67.9	0.3	—	24.7	0.4	0.1	0.5	5.8	0.4	
	21	72.1	0.3	—	20.9	0.4	0.2	0.4	5.4	0.3	
	22	69.8	0.3	—	22.4	0.4	0.3	0.5	5.9	0.4	
	24	75.4	0.3	—	18.5	0.4	0.3	0.4	4.3	0.4	
	25	70.3	0.3	—	23.3	0.4	0.1	0.4	4.8	0.4	
Vitreous china		70.1	0.3	—	25.0	0.7	0.2	0.1	2.6	1.1	[28]
Porcelain	S5	66.8	0.5	—	28.4	0.4	—	0.3	0.9	2.6	[29]
	S6	66.4	0.6	—	28.7	0.5	—	0.3	0.8	2.7	
	S7	66.2	0.6	—	28.7	0.5	0.3	0.3	0.8	2.6	

Material	Sample	SiO_2	TiO_2	ZrO_2	Al_2O_3	Fe_2O_3	MgO	CaO	Na_2O	K_2O	Ref.
Hard porcelain	S1	50.9	0.5	—	44.7	0.4	—	0.2	—	3.3	[29]
	S2	50.2	0.6	—	45.6	0.4	—	0.2	—	3.0	
	S3	49.1	0.5	—	46.3	0.4	—	0.2	—	3.6	
	S4	46.5	0.5	—	47.9	0.5	—	0.2	0.5	3.7	

Table S2. Phase composition of porcelain stoneware bodies in comparison with hard porcelain, porcelain, and vitreous china batches.

Material	Sample	Corundum	Quartz	Mullite	Albite	Orthoclase	Rutile	Zircon	Glass	Ref.
Porcelain Stoneware	1200	—	28.6	12.6	—	—	—	—	58.7	[26]
	STD.6	—	17.0	13.0	2.0	—	—	3.0	65.0	[10]
	QF	—	21.0	12.0	2.5	—	—	2.5	62.0	
	QM	—	17.0	11.0	2.0	—	—	2.0	68.0	
	KM	—	14.0	11.0	2.0	—	—	3.0	70.0	
	QFKM	—	18.0	13.0	2.5	—	—	2.5	64.0	
	QMKM	—	16.0	12.0	3.0	—	—	2.0	67.0	
	STD.6	—	16.0	10.0	3.4	—	—	2.0	68.6	
	STD.9	—	16.0	11.0	3.0	—	—	2.0	68.0	
	KM	—	13.0	11.0	3.0	—	—	2.0	71.0	
	QFKM	—	14.5	10.0	1.6	—	—	2.0	71.9	
	QMKM	—	15.0	11.0	4.0	—	—	2.0	68.0	
	Na	—	14.1	10.0	—	—	—	—	75.9	This Work
	NaB	—	15.2	10.3	—	—	—	—	74.5	
	AT	—	16.8	6.4	—	—	0.1	—	76.7	
	ATP	—	20.2	5.7	1.0	—	0.2	—	72.9	
	NaK	—	15.1	8.7	—	—	—	—	76.2	
	K	—	15.4	8.8	—	—	—	—	75.8	
	KB	—	15.1	16.8	—	—	—	—	68.1	
	2	—	12.1	13.9	0.8	0.3	—	—	72.8	[27]
	7	—	10.1	12.6	0.6	0.6	—	—	76.0	
	21	—	21.7	11.1	1.1	0.5	—	—	65.6	
	22	—	14.1	12.2	1.1	0.5	—	—	72.0	
	24	—	29.8	11.2	1.0	0.4	—	—	57.6	
	25	—	21.1	16.3	1.4	0.7	—	—	60.5	
Vitreous China	0-1240-50	—	28.3	16.5	3.4	—	—	—	51.8	[28]
	20-1240-50	—	26.7	18.0	—	—	—	—	55.3	
	40-1240-50	—	23.6	17.7	—	—	—	—	58.7	
	60-1240-50	—	22.6	18.0	—	—	—	—	59.4	
	80-1240-50	—	21.4	19.5	—	—	—	—	59.1	
	0-1280-50	—	24.2	17.4	—	—	—	—	58.4	
	20-1280-50	—	24.1	17.7	—	—	—	—	58.2	
	40-1280-50	—	22.3	17.6	—	—	—	—	60.1	
	60-1280-50	—	19.9	17.6	—	—	—	—	62.5	
	80-1280-50	—	19.5	17.6	—	—	—	—	62.9	

Material	Sample	Corundum	Quartz	Mullite	Albite	Orthoclase	Rutile	Zircon	Glass	Ref.
Vitreous china	0-1240-18	—	24.7	17.8	3.5	—	—	—	54.0	[28]
	20-1240-18	—	19.8	17.3	—	—	—	—	62.9	
	40-1240-18	—	18.7	17.6	—	—	—	—	63.7	
	60-1240-18	—	16.3	17.1	—	—	—	—	66.6	
	80-1240-18	—	15.0	17.5	—	—	—	—	67.5	
	0-1280-18	—	21.8	18.2	—	—	—	—	60.0	
	20-1280-18	—	18.5	17.6	—	—	—	—	63.9	
	40-1280-18	—	14.3	16.8	—	—	—	—	68.9	
	60-1280-18	—	13.9	16.9	—	—	—	—	69.2	
	80-1280-18	—	13.0	16.4	—	—	—	—	70.6	
Porcelain	S5	8.8	23.0	5.4	0.1	—	—	—	62.7	[29]
	S6	8.9	23.8	5.9	—	—	0.3	—	61.2	
	S7	8.5	22.6	5.1	—	—	0.2	—	63.6	
Hard porcelain	S1	33.2	12.1	3.3	—	—	0.2	—	51.1	[29]
	S2	34.2	10.8	4.0	—	—	0.2	—	50.7	
	S3	35.3	8.5	5.0	—	—	0.1	—	51.1	
	S4	34.9	8.4	5.8	—	—	—	—	50.9	

Table S3. Chemical composition of silicate melts taken into account to assess the Giordano and coworkers' [19] and Fluegel's [17] models used to calculate the viscosity at high temperature.

Sample	SiO_2	TiO_2	Al_2O_3	FeO	MgO	CaO	Na_2O	K_2O	MnO	P_2O_5	Ref.
IGC	60.7	0.3	19.2	3.4	0.3	2.1	5.3	6.3	0.2	0.1	[33]
MNV	63.9	0.3	17.1	2.9	0.2	1.8	5.7	6.8	0.1	0.1	[33]
MST	60.7	0.6	18.3	6.4	2.6	7.1	3.6	0.9	0.2	0.0	[34]
CI_OF	68.8	0.2	12.6	3.2	1.2	3.4	4.0	6.2	0.1	0.0	[34]
MDV	79.4	0.2	9.9	1.9	1.6	2.4	0.5	3.4	0.0	0.0	[34]
G.2000	60.5	0.6	18.8	3.3	0.4	0.7	9.8	5.5	0.2	0.1	[32]
AMS-B1	61.3	0.4	18.4	3.5	0.7	3.0	4.6	8.0	0.1	—	[35]
AMS-D1	60.9	0.4	18.3	3.9	0.9	3.0	4.1	8.5	0.1	—	[35]
Trachyte	64.5	0.5	16.7	0.0	2.9	5.4	6.7	3.4	—	—	[36]
Phonolite	58.8	0.8	19.4	0.0	1.9	2.4	9.3	7.4	—	—	[36]

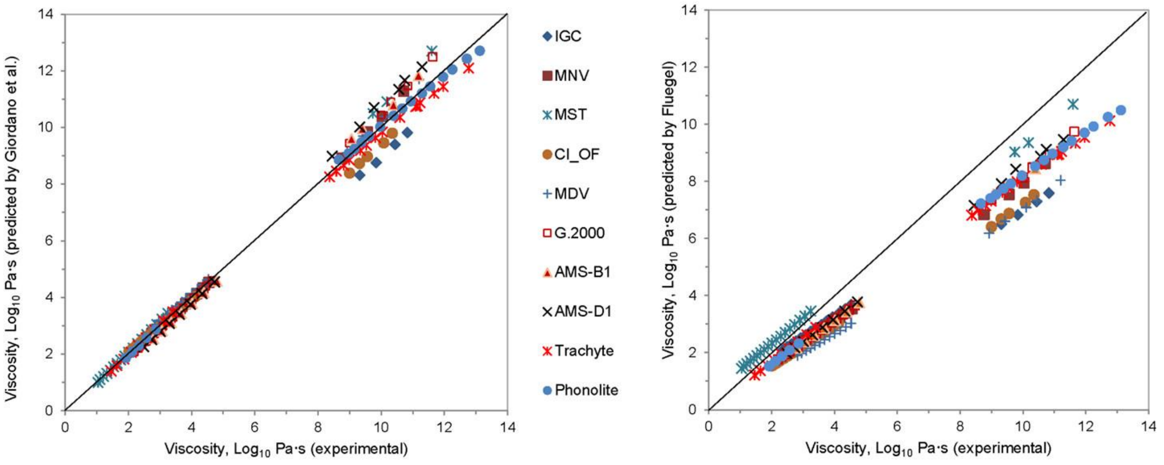

Figure S1 Viscosity predicted versus experimental: Giordano and coworkers' model (left) and Fluegel's model (right). The viscosity interval of interest for porcelain-like materials is in between 4 and 6 Log_{10} Pa·s. IGC [33], MNV [33], MST [34], CI_OF [34], MDV [34], G.2000 [32], AMS-B1 [35], AMS-D1 [35], Trachyte [36], Phonolite [36].

Table S4. Comparison between the calculated and the experimentally measured viscosity values of silicate melts.

Sample	Temp. °C	Exp. Log_{10} Pa·s	Giordano Log_{10} Pa·s	Fluegel Log_{10} Pa·s	Samp le	Temp. °C	Exp. Log_{10} Pa·s	Giordano Log_{10} Pa·s	Fluegel Log_{10} Pa·s
IGC	1496	2.37	2.39	2.10	MDV	1643	2.83	3.03	1.89
[33]	1471	2.49	2.52	2.20	[34]	1619	2.96	3.15	1.98
	1446	2.63	2.65	2.30		1594	3.10	3.28	2.07
	1422	2.77	2.79	2.40		1569	3.24	3.41	2.16
	1397	2.92	2.93	2.51		1545	3.38	3.54	2.25
	1373	3.08	3.07	2.62		1520	3.53	3.68	2.35
	1348	3.24	3.23	2.73		1496	3.69	3.83	2.45
	1323	3.40	3.39	2.86		1471	3.84	3.98	2.56
	1299	3.58	3.55	2.98		1446	4.01	4.13	2.67
	1274	3.76	3.73	3.11		1422	4.18	4.30	2.78
	1249	3.94	3.91	3.24		1397	4.36	4.46	2.90
	1225	4.14	4.09	3.38		1373	4.53	4.64	3.02
	1200	4.34	4.29	3.53		920	9.43	9.69	6.60
	1176	4.54	4.49	3.68		882	10.11	10.37	7.09
	861	9.32	8.32	6.50		865	10.45	10.71	7.33
	836	9.84	8.76	6.83		818	11.20	11.70	8.04
	803	10.44	9.40	7.29		955	8.92	9.11	6.19
	783	10.83	9.82	7.59	MST	1619	1.04	1.00	1.44
MNV	1496	2.50	2.48	2.00	[34]	1594	1.11	1.10	1.52
[33]	1471	2.62	2.61	2.10		1569	1.21	1.21	1.61
	1446	2.75	2.74	2.20		1545	1.32	1.31	1.70
	1422	2.89	2.87	2.29		1520	1.43	1.43	1.80
	1397	3.03	3.01	2.40		1496	1.53	1.55	1.90
	1373	3.18	3.15	2.50		1471	1.65	1.67	2.00
	1348	3.33	3.30	2.62		1446	1.76	1.80	2.10
	1323	3.49	3.46	2.73		1422	1.89	1.93	2.21

MNV	1299	3.65	3.62	2.85
[33]	1274	3.82	3.79	2.98
	1249	3.97	3.96	3.11
	1225	4.17	4.14	3.24
	1200	4.36	4.33	3.39
	1176	4.55	4.53	3.53
	817	8.76	8.92	6.84
	769	9.56	9.85	7.54
	744	10.03	10.39	7.95
	706	10.71	11.28	8.63
CI_OF	1594	2.02	2.09	1.55
[34]	1569	2.14	2.20	1.63
	1545	2.26	2.31	1.71
	1520	2.39	2.43	1.80
	1496	2.52	2.55	1.89
	1471	2.66	2.67	1.99
	1446	2.80	2.80	2.09
	1422	2.94	2.94	2.19
	1397	3.10	3.08	2.29
	1373	3.25	3.22	2.40
	1348	3.42	3.37	2.52
	1323	3.59	3.53	2.64
	1299	3.76	3.69	2.76
	1274	3.94	3.86	2.89
	1249	4.13	4.04	3.03
	856	9.00	8.39	6.41
	836	9.31	8.74	6.69
	823	9.56	8.98	6.88
	797	10.08	9.46	7.26
	780	10.35	9.80	7.53
AMS-B1	1446	2.79	2.55	2.17
[35]	1397	3.06	2.82	2.38
	1348	3.35	3.12	2.61
	1299	3.67	3.44	2.86
	1249	4.02	3.79	3.14
	1200	4.39	4.17	3.43
	1151	4.80	4.58	3.75
	785	9.06	9.60	7.56
	768	9.41	9.96	7.82
	733	10.39	10.80	8.45
	694	11.18	11.83	9.21
Trachyte	678	12.77	12.10	10.12
[36]	700	11.96	11.44	9.54
	709	11.67	11.21	9.34
	721	11.23	10.87	9.05
	725	11.14	10.76	8.95
	726	11.09	10.74	8.93
	741	10.59	10.35	8.60
	763	10.04	9.84	8.16
	772	9.82	9.64	7.99
	784	9.53	9.38	7.76
	793	9.34	9.19	7.60
MST	1397	2.01	2.07	2.33
[34]	1373	2.15	2.21	2.45
	1348	2.29	2.36	2.57
	1323	2.43	2.52	2.70
	1299	2.58	2.69	2.84
	1274	2.73	2.86	2.98
	1249	2.90	3.04	3.13
	1225	3.06	3.23	3.29
	1200	3.25	3.44	3.45
	753	9.74	10.49	9.03
	740	10.18	10.91	9.35
	689	11.60	12.70	10.70
AMS-D1	1496	2.49	2.26	1.97
[35]	1446	2.74	2.52	2.18
	1397	3.01	2.79	2.39
	1348	3.30	3.08	2.63
	1299	3.62	3.40	2.88
	1249	3.96	3.75	3.16
	1200	4.33	4.13	3.45
	1151	4.73	4.55	3.77
	814	8.45	8.99	7.15
	765	9.32	10.02	7.92
	737	9.77	10.70	8.42
	712	10.56	11.34	8.88
	700	10.75	11.67	9.12
	684	11.29	12.14	9.46
G.2000	1500	2.20	2.08	1.77
[32]	1475	2.32	2.19	1.86
	1450	2.44	2.31	1.94
	1425	2.57	2.44	2.04
	1400	2.70	2.57	2.13
	1375	2.83	2.70	2.23
	1350	2.97	2.84	2.34
	1325	3.11	2.99	2.45
	1300	3.26	3.14	2.56
	1275	3.42	3.30	2.68
	1250	3.57	3.46	2.80
	1225	3.74	3.63	2.92
	1200	3.91	3.81	3.06
	1175	4.07	3.99	3.20
	1150	4.27	4.19	3.34
	1125	4.46	4.39	3.50
	1100	4.65	4.60	3.66
	615	11.63	12.50	9.74
	651	10.85	11.47	8.93
	673	10.31	10.90	8.49
	692	10.00	10.45	8.13
	737	8.99	9.45	7.36
Phono	616	13.12	12.71	10.49
lite	625	12.71	12.43	10.25
[36]	638	12.25	12.05	9.92
	647	11.96	11.79	9.70

Trachyte [36]	809	8.99	8.85	7.31	Phono lite [36]	659	11.55	11.45	9.42
	819	8.81	8.67	7.16		668	11.29	11.21	9.20
	830	8.58	8.46	6.98		679	10.94	10.93	8.97
	840	8.37	8.26	6.81		689	10.67	10.67	8.75
	1655	1.46	1.39	1.21		700	10.39	10.41	8.53
	1606	1.64	1.59	1.37		717	9.97	10.03	8.20
	1554	1.86	1.82	1.54		732	9.62	9.70	7.92
	1503	2.09	2.05	1.73		742	9.38	9.48	7.74
	1453	2.32	2.31	1.94		754	9.15	9.25	7.55
	1405	2.56	2.57	2.14		762	8.97	9.09	7.41
	1355	2.83	2.85	2.37		773	8.66	8.86	7.22
	1305	3.12	3.17	2.63		1542	1.92	1.86	1.53
	1258	3.41	3.49	2.88		1493	2.12	2.09	1.70
						1449	2.33	2.30	1.87
						1396	2.57	2.57	2.09
						1344	2.86	2.86	2.32
